# Randomized Controlled Trial of Enteral Vitamin D Supplementation (ViDES) in Infants <28 Weeks Gestational Age or <1000 Grams Birth Weight: Study Protocol

**DOI:** 10.21203/rs.3.rs-4049246/v1

**Published:** 2024-06-25

**Authors:** Mar Romero-Lopez, Jon E Tyson, Mamta Naik, Claudia Pedroza, Lindsay F Holzapfel, Elenir Avritscher, Ricardo Mosquera, Amir Khan, Matthew Rysavy

**Affiliations:** The University of Texas Health Science Center at Houston; The University of Texas Health Science Center at Houston; Children’s Memorial Hermann Hospital; The University of Texas Health Science Center at Houston; The University of Texas Health Science Center at Houston; The University of Texas Health Science Center at Houston; The University of Texas Health Science Center at Houston; The University of Texas Health Science Center at Houston; The University of Texas Health Science Center at Houston

**Keywords:** Vitamin D, extremely preterm infant, randomized controlled trial, bronchopulmonary dysplasia, sepsis, neurodevelopmental disorders, osteopenia of prematurity, bayesian analyses

## Abstract

**Background:**

Vitamin D is necessary to develop healthy lungs and other organs early in life. Most infants born before 28 weeks’ gestation have low vitamin D levels at birth and a limited intake during the first month. Enteral vitamin D supplementation is inexpensive and widely used. The appropriate supplementation regimen for extremely preterm infants is controversial, and the effect of different regimens on their blood levels and outcomes is unclear.

**Methods:**

Randomized, blinded comparative effectiveness trial to compare two vitamin D supplementation regimens for inborn infants <28 weeks gestation or <1000 grams birth weight at a large academic center in the United States.

Infants are stratified by birth weight and randomized within 96 hours after birth to either routine supplementation (400 IU/day with established feedings) or increased supplementation (800 IU/day with any feedings) during the first 28 days after birth.

We hypothesize that the higher and early vitamin D dose (800 IU/d with early feeding) compared to placebo plus routine dose (400 IU/d with established feeding) will substantially increase total 25-hydroxyvitamin D3 levels measured as state-of-art at one month, reduce respiratory support at 36 weeks’ postmenstrual age (on an ordinal scale predictive of later adverse outcomes) and improve or at least not worsen other important secondary outcomes. The infants in the study will follow up at 22–26 months’ corrected age (~2 years) with blinded certified examiners to evaluate neurodevelopmental outcomes.

The sample size of a minimum of 180 infants provides >90% power to detect a >95% posterior probability of a 33% increase in serum 25-hydroxy vitamin D3 and >80% power to detect a >80% posterior probability of a relative risk decrease of 20% of reducing respiratory support by intention-to-treat Bayesian analyses using a neutral prior probability.

**Discussion:**

Our study will help clarify the uncertain relationship of vitamin D supplementation and its associated serum metabolites to clinical outcomes of extremely preterminfants. Confirmation of our hypotheses would prompt reconsideration of the supplementation regimens used in extremely preterm infants and justify a large multicenter study to verify the generalizability of the results.

**Trial registration:**

ClinicalTrials.gov registered on July 14, 2022 (NCT05459298)

## Introduction

### Background and rationale {6a}

Vitamin D is critical for many essential physiological processes. In early life, it is important for bone formation and calcium metabolism [1], regulation of immune and inflammatory responses[2], and development and maturation of the lungs (improving alveolarization, proliferation of vascular endothelial cells, and synthesis of surfactant)[3–5].It may also have direct or indirect importance to brain development [6].

Because vitamin D is transferred mainly to the fetus during the third trimester, preterm infants are born with low vitamin D levels and stores [5,7,8]. In a recent meta-analysis, low vitamin D levels relative to current norms during infancy were identified in 70% of the preterm infants with a mean gestational age (**GA**) of 29 weeks [9]. During the first month after birth, low vitamin D levels persist in extremely preterm (**EP**) infants due to the low vitamin D content in human milk, prolonged or recurrent feeding intolerance common in EP infants, and administration of medications like caffeine and corticosteroids[10].

Unfortunately, the appropriate vitamin D supplementation for EP infants is uncertain, and vitamin D recommendations range from 200–1000 IU/day [11–13]. Supplementation with 400 IU/day as recommended by the American Academy of Pediatrics [12] is not likely to correct the vitamin D deficiency for EP at 4 weeks after birth by current norms. A recent cohort study of 301 infants (115 infants <1000g birth weight [**BW**]), 80% had low 25-hydroxy vitamin D3 (**25-OH-D**_**3**_) level 4 weeks after birth, and the infants less than 1000 g did not develop sufficient serum levels of vitamin D until 12 weeks [14].

To our knowledge, only one clinical trial has compared lower (0–400 IU/day) and higher supplementation (800–1000 IU/day) in EP infants [15]. Although the differences in outcomes did not reach statistical significance, the findings with the higher supplementation regimen were compatible with a 28% reduction in bronchopulmonary dysplasia (**BPD**) or death at 36 weeks postmenstrual age (**PMA**) (Relative Risk [**RR**] 0.72 [95% CI 0.37–1.41]) and a 40% reduction in neurodevelopmental impairment (NDI) at 2 years (RR 0.60 [95% CI: 0.3–1.2]) [15,16].

While other potential benefits of higher dose vitamin D supplementation have not been adequately studied, a meta-analysis of 12 small trials identified a significant increase in length and head circumference and several measures of immune function when preterm infants were supplemented with 800–1000IU/day compared to the 400 IU/day [17].

Appropriate supplemental vitamin D would be a low-cost nutritional intervention available worldwide that could reduce respiratory support, BPD, infections, osteopenia, poor growth, and/or NDI in EP infants, and the long-term adverse effects of these disorders for these babies and their families [18].

Additional clinical trials to assess increased vitamin D supplementation for EP infants are clearly needed, given the low cost of vitamin D, the wide variety of potential benefits, and the high prevalence of adverse outcomes in these infants.

We will analyze our single-center trial using conservative Bayesian analyses to directly assess the probability of clinical benefits from higher dose supplementation and to avoid the limitations of traditional frequentist analyses, particularly for studies with a limited sample size [19–23].

### Objectives {7}

The objectives are to determine if higher dose supplementation (800 IU/day with any feedings) compared to our routine supplementation regimen (400 IU/day with established feedings) for EP infants during the first 28 days after birth will:

- Increase the level of 25-OH-D_3_ one month after birth by more than 33%
- Reduce the need for respiratory support at 36 weeks’ PMA with a probability of more 80% (using the ordinal outcome described below and Bayesian analyses)
- Improve or at least not worsen other secondary outcomes (bronchopulmonary dysplasia; duration of respiratory support; hospital stay; infections; fractures; growth and development through 2 years and death or individual morbidities).

### Trial design {8}

Randomized, blinded, parallel-group comparative effectiveness trial with a 1:1 allocation ratio. The trial is designed as a superiority trial.

## Methods: Participants, interventions and outcomes

### Study setting {9}

The study will take place at the neonatal intensive care unit (**NICU**) at Children’s Memorial Hermann Hospital in Houston, Texas, one of the largest NICU in the U.S., with >1400 admissions and >100 infants <29 weeks’ gestation per year.

### Eligibility criteria {10}

#### Inclusion criteria:

<28 weeks’ gestation (best obstetric estimate) or <1000 g BW; age <96 h at enrollment; inborn.

#### Exclusion criteria:

GA ≥32 weeks; major anomaly; overt congenital nonbacterial infection; severe illness or immaturity that the attending neonatologist judges intensive care to be unjustified; or prenatal diagnosis of cystic fibrosis.

### Who will take informed consent? {26a}

Trained research personnel approach the mother or authorized legal guardian of eligible infants within 96 hours after birth and provide a copy of the informed consent form, allowing sufficient time to make an informed decision.

### Additional consent provisions for collection and use of participant data and biological specimens {26b}

In the event of withdrawal from the study, the parents will be asked for their consent to continue the collection of electronic data. The consent form includes the collection of blood for vitamin D metabolites.

### Interventions

#### Explanation for the choice of comparators {6b}

As may be typical [24], depending on their weight, age, and feeding tolerance, our patients in the routine care group receive a total vitamin D intake from all sources of ~160 – 600 IU/day during the first month after birth. Multivitamins, providing 160 IU/kg/day of vitamin D, are added to the parenteral nutrition from day 2 after birth. Almost all infants are fed human milk (mothers’ or donors’ human milk) that contains minimal vitamin D. When the infant tolerates 80 ml/kg/day, feedings are fortified up to 24 kcal/oz with bovine-derived human milk forti er, and the parenteral multivitamins are discontinued. Enteral supplementation of 400 IU/day of vitamin D3 (cholecalciferol) is started when feedings reach ~120 ml/kg/day. We routinely increase fortified feedings to 160 ml/kg/day to support growth, providing ~190 IU/kg/day of vitamin D. Administering an inactive placebo (normal saline) is justified due to the uncertainty surrounding the optimal dosage of vitamin D supplementation for EP infants during the initial month after birth. (Table 1).

#### Intervention description {11a}

The intervention group is supplemented with 800 IU/day with any feeding for the first 28 days after birth. The 800 IU/day is divided into 4 doses/day and are administered every 6 hours with feedings to reduce osmolality and provide a more uniform intake that may be better tolerated, absorbed, and utilized [15]. (Table 1).

#### Criteria for discontinuing or modifying allocated interventions {11b}

Two independent blinded-to-treatment group investigators review the electronic medical records of the study infants daily during their admission to the NICU. The study drug will be discontinued if vitamin D toxicity is suspected, at the attending physician’s discretion, and for the period the infant is not receiving feeding during the intervention period.

#### Strategies to improve adherence to interventions {11c}

Extensive education was provided before the study started. A laminated card with a QR code linking to study information and the team’s contact details is placed on the patient’s bedside. The research study closely monitors the patient to ensure protocol compliance. A missed dose can be given with the next feed, with a maximum of 2 doses together, and must be administered within 24 hours. Before the vitamin D level collection, the research team calls the bedside nurse to provide a reminder of the blood sample collection time.

#### Relevant concomitant care permitted or prohibited during the trial {11d}

No more than 400 IU/day of vitamin D enteral supplementation as part of routine care is allowed in any group during the first 28 days after birth. The infant will only receive the study drug (extra vitamin D or placebo) if receiving feedings. The blood collection for enrolled infants is only collected with usual laboratory testing, and no additional venipuncture for the study will occur.

#### Provisions for post-trial care {30}

Infants will be provided with comprehensive care in the high-risk follow-up program that includes treatment for acute and chronic illnesses from subspecialists and primary care providers available in the clinic 40 hours per week and by telephone at all hours. This program includes multiple measures to promote prompt and effective outpatient and inpatient care, and it has been shown in randomized trials to increase parent satisfaction, achieve high follow-up rates, and reduce serious illnesses (prolonged hospitalizations, pediatric intensive care unit admissions, or death) [25,26].

#### Outcomes {12}

The primary outcome is 25-(OH)D_3_ levels at one month after birth.

The secondary outcomes are indicators of likely or plausible effects of vitamin D supplementation on the function or structure of the lungs, bones, immune system, and brain, including:

- Respiratory support required at 36 weeks PMA using an ordinal scale (Table 2).

Nasal cannula [**NC**]

Severity graded according to the mode of support at 36 weeks PMA, irrespective of oxygen supplementation needs

- Duration of respiratory support up to 2 years corrected age (**CA**), steroid treatment to decrease respiratory
- support, pulmonary hypertension at 36 weeks PMA, and wheezing at 2 years.
- Weight, length, and head circumference at birth, 1 month, 36 weeks’ PMA, and at 2 years’ CA. Fractures identified before NICU discharge and up to 2 years of CA. Serum calcium, phosphorus, and alkaline phosphatase levels during the first 28 days after birth and at 36 weeks’ PMA.
- Bayley^™^ Scales of Infant and Toddler Development, fourth edition (**BSID-IV**) neurodevelopmental assessments and NDI at 2 years’ CA.
- Hospital-acquired sepsis, length of stay and readmissions.
- Serum concentrations of 25-OH-D_3_, 25-OH-D_2,_ 3-epi-25-OH-D_3,_ and 24,25-(OH)_2_D_3_ by Liquid Chromatography-tandem Mass spectrometry.

Other outcomes are important differences between groups in other major clinical diagnoses are unexpected with our sample size and will be considered hypothesis-generating. We will report grade III-IV intraventricular hemorrhage, periventricular leukomalacia, surgically treated necrotizing enterocolitis, spontaneous intestinal perforation, stage III-V retinopathy of prematurity, and patent ductus arteriosus.

#### Participant timeline {13}

Screening for infants meeting inclusion criteria is performed daily. The infant enrolled in the study will be randomized between 24 and 96 hours after birth.

The first sample of blood is obtained soon after consent is obtained. The intervention will be stopped 28 days after birth; a second blood sample is collected with the next usual laboratory test after finishing the intervention (around 1 month postnatal), and the third sample at 36 weeks or before discharge, whichever occurs first.

The study intervention (placebo vs. extra vitamin D) is given if the infant is feeding within the first 28 days after birth and the vitamin D intake, relevant laboratory results, respiratory support, and growth parameters are obtained from the medical records during the NICU stay and during a 2-year follow-up appointment.

**Figure 1:**
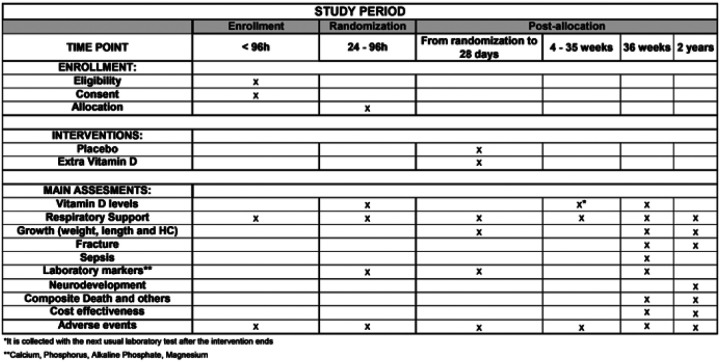
Study schedule

Standard follow-up assessments [27] will be conducted at 22–26 months’ adjusted age, including neurologic examination and neurodevelopmental and behavioral assessment. Additionally, we will collect data on long-term respiratory outcomes, including recurrent wheezing (modified International Study of Asthma and Allergies in Childhood questionnaire[28]) and respiratory infections, to evaluate early childhood pulmonary health. (Fig.1)

#### Sample size {14}

Assuming a mean 25-OH-D_3_ = 25 (Standard Deviation [SD] =12) ng/ml based on the data of a recent study[14] with a similar population and vitamin D supplementation to our routine care, 74 infants (37 in each group) affords ≥90% Bayesian power (with a >95% posterior probability) to identify an increase in serum 25-OH-D_3_ in the intervention group, assuming a true increase of 33%. We will assess the largest sample size feasible during the study period of three years (≥180 infants) to maximize precision in the estimate of effect and power for other outcomes based on our annual admission rate of infants that will qualify for the study, assuming a consent rate of 60%. Using a neutral Bayesian prior probability (RR =1.0; 95% CrI, 0.3–3.3), 180 total patients afford 80% power to identify a >80% posterior probability (intervention arm) of reducing respiratory support (based on the described ordinal outcome) assuming a true RR decrease of 20% (RR=0.80) and 90% power if the true RR decrease is 25%.

#### Recruitment {15}

A dedicated and highly skilled research team is available 24 hours per day, seven days per week, to maximize recruitment, which is expected to end by July 2025.

### Assignment of interventions: allocation

#### Sequence generation {16a}

The sequence is generated 1:1 with variable block size (4–6) using research electronic data capture (REDCap). Infants are stratified by BW (<750 g or ≥750 g) and assigned by enrollment order consecutively. Multiples are randomized separately.

#### Concealment mechanism {16b}

The study statistician provided the allocation sequence to the investigational drug pharmacy (**IDP**), matching the assigned code with the intervention (placebo vs vitamin D). After patient randomization, an encrypted email is sent by REDCap to IDP with name, medical record number, and assigned code.

#### Implementation {16c}

The research team is notified when IDP receives the email to prepare the intervention, contact the medical and nursing team regarding the study’s first dose, and deliver support material to facilitate adherence to the protocol.

### Assignment of interventions: Blinding

#### Who will be blinded {17a}

Parents, clinical staff, and research personnel are blinded to the study arm. Only the statistician and IDP personnel know the allocation of the study patients. Our IDP ensures that both arms’ quality control and regulatory compliance. Placebo and vitamin D (Enfamil Di-vi-sol^®^ is a clear, odorless solution) have the same volume and appearance.

#### Procedure for unblinding if needed {17b}

If there are concerns for vitamin D toxicity, the study drug or placebo will be stopped without unblinding the NICU staff and research team. The study statistician, NICU medical director, and the Institutional Review Board (**IRB**) will be notified of any infant considered to have signs suggestive of vitamin D toxicity and any infant with a serious adverse event by the National Institute of Health definition [29].

### Data collection and management

#### Plans for assessment and collection of outcomes {18a}

The study data are largely obtained from the patients’ electronic medical record by the research team. Automatic data checks identify values outside the expected range at data entry to prompt verification that the data are correct.

Head circumference (assessed using tape) and length (assessed using a length board) are measured by research team members trained to achieve highly reliable measurements at one month and 36 weeks’ PMA.

Neurodevelopmental assessment, including BSID-IV at 22–26 months corrected age, Gross Motor Function Classification System, and hearing and vision evaluation, will be performed by certified examiners by the National Institute of Child Health and Human Development Neonatal Research Network [30]. Moderate or severe NDI is defined as a composite of neurologic, developmental, hearing, and vision status. Using the BSID-IV, a moderate delay in the cognitive domain is defined as 70–84, severe delay < 70, and profound delay ≤ 54. Other assessments include a Gross Motor Function Classification Score of ≥2, hearing impairment as bilateral permanent hearing loss that hinders the child’s ability to comprehend examiner directions, with or without amplification, and vision impairment characterized by bilateral acuity less than 20–200 with amplification.

Additionally, we will collect data on long-term respiratory outcomes, including recurrent wheezing (modified International Study of Asthma and Allergies in Childhood questionnaire [28]) and respiratory infections, to evaluate early childhood pulmonary health.

Vitamin D metabolites determination: A total maximum blood volume of <1 ml is obtained for the entire study duration (3 samples of 0.3 ml of whole blood), with daily clinical labs. Specimens are placed in a gold-top microtainer, stored in the research refrigerator until separation of serum in the next 6 hours, and frozen at −80F before shipping in batches for analysis to King’s University College in Ontario, Canada.

Vitamin D metabolites are extracted using liquid-liquid extraction (**LLE**) and immunoextraction [31,32] and subjected to liquid chromatography-tandem mass spectrometry analysis using Waters ACQUITY UPLC/Xevo TQ-S instruments. The focus will be on abundant vitamin D metabolites, including 25-OH-D_3_, 25-OH-D_2_ and 3-epi-25-OH-D_3_.

#### Plans to promote participant retention and complete follow-up {18b}

As in other trials in our NICU, we expect very few parents to want to withdraw their infant from the trial. If they do, we will stop the study drug and blood collection but ask the parents to allow us to include the infant’s clinical outcomes in our analyses. This approach, with a strong and innovative follow-up program with expertise in clinical trials with follow-up interventions [26,33,34], minimizes loss to follow-up and helps maximize the proportion of randomized infants included in our intention-to-treat analyses. Based on our experience with other studies, we expect to identify outcomes at 2 years for ≥80% of EP infants enrolled in this trial.

#### Data management {19}

The data is entered and stored in a protected system (REDCap) with quality data checks (automated range checks for data values). Before performing the interim and final analyses, the statistician will perform quality checks and communicate with the research team if problems are encountered.

#### Confidentiality {27}

REDCap is a secure online database, with minimal risk of loss of confidentiality. Personal identifiers will be removed from the information and samples collected in this study. A study number will be used to identify the patient for data analysis.

#### Plans for collection, laboratory evaluation, and storage of biological specimens for genetic or molecular analysis in this trial/future use {33}

The collected samples will be sent to Dr. Jones laboratory at King’s University College in Ontario,Canada, with the intent to analyze the vitamin D metabolites of the patients enrolled in the study. After a maximum of 3 years, they will be discarded.

### Statistical methods

#### Statistical methods for primary and secondary outcomes {20a}

Intention-to-treat Bayesian analyses [22] will be performed using generalized linear mixed models for all outcomes. Covariates for the primary and secondary outcomes will include the treatment group and BW (<750 g, ≥750 g). All models will also include a random effect for family (to account for within-family correlation). The primary outcome of the 25-OH-D3 level will be analyzed with a linear mixed regression model, and the ordinal respiratory outcome will be analyzed with an ordinal logistic model. Binary outcomes will be analyzed with logistic regression, and data will be counted with a Poisson or negative binomial regression model. Priors will be centered at an odds ratio (RR for count data) of 1.0 (indicative of no difference between treatment groups with a 95% credible interval (**CrI**) of 0.3–3.3 and in the log OR scale a Normal distribution with a mean of 0 and variance of 0.70). A Normal(0, SD=10) prior will be used for the intercept term, a Normal(0,1) for all other variables in the model, and half-Normal(0,1) for the SD of the random effect. We will report posterior medians and 95% CrI for group differences, ORs, RRs, and each outcome’s posterior probability of benefit.

All analyses will be conducted in R software latest version. Bayesian models will be fit using Markov chain Monte Carlo (MCMC) methods implemented in Stan via R packages ‘rstanarm’ and ‘brms’. Each model will be fit using three MCMC chains. Each chain will have at least 5,000 burn-in samples and at least 20,000 additional iterations. Convergence of MCMC chains will be assessed by visual comparisons of the trace plots for all parameters from the 3 chains generated for each model. Quantitative checks including the Geweke and Gelman/Rubin tests will be used to ensure convergence to the posterior distribution for all analyses.

#### Interim analyses {21b}

A single interim analysis using the same statistical approach as for the final analysis will be performed when half the patients have reached 36 weeks’ PMA. The study will be stopped at this interim if 1) there are persistent biochemical signs of vitamin D toxicity (hypercalcemia Ca level >11mg/dL, ionized Ca >1.55 nMol/L with phosphorus >4mg/dL and 25-OH-vitamin D level >150ng/mL) that are otherwise unexplained and are seen in multiple infants or 2) there is a > 95% probability that extra vitamin D increases by more than 15 % (i.e.., risk ratio >1.15) the rates of death or individual major morbidities unlikely to be related to vitamin D supplementation, specifically BPD, hospital-acquired infections, necrotizing enterocolitis, or spontaneous intestinal perforation by themselves or as a composite outcome with death. Because of the importance of the outcomes at 2 years CA, enrollment will be stopped for benefit at interim analysis only if there is a ≥98% probability that vitamin D reduces the ordinal respiratory support outcome at 36 weeks’ PMA.

#### Methods for additional analyses (e.g. subgroup analyses) {20b}

Subgroup analyses of both RR/odds ratio and risk difference will be performed to assess effect modification by BW, gestational age, sex, race, infants who receive at least 80% of the possible doses (infants with minimal time when feedings were discontinue) and baseline 25-OH-D_3_ level (each assessed in one variable at a time analyses) and of different risk strata (assessed using multiple variables). Bayesian hierarchical models with interaction terms between the treatment group indicator and the prespecified potential modifiers will be used. The conservative Bayesian approach of Dixon and Simon [35] will be applied as it allows for a priori specification of how likely (or unlikely) it is for subgroup differences to be present, and to shrink the subgroup estimates to the overall mean treatment effect. The prior distributions for main effects will be the same for subgroup analyses as for the primary endpoint analysis. A Normal(0, SD=0.7) will be used for interaction terms. Point estimates of treatment effect and 95% CrI for each subgroup will be reported along with probability of benefit using a forest plot.

#### Methods in analysis to handle protocol non-adherence and any statistical methods to handle missing data {20c}

Per-protocol analyses will be conducted for the primary and major secondary outcomes using the same methods as for the interim analyses. The per-protocol analysis dataset will include all infants who received the randomized intervention (routine care or intervention).

If missing data occurs, it will be assumed to be noninformative (missing at random), and it will be imputed under a Bayesian framework by simulating from the posterior predictive distribution.

#### Plans to give access to the full protocol, participant level-data and statistical code {31c}

We will grant public access to the full protocol, participant-level dataset, and statistical code upon request within one year after the publication of the study. We will foster collaboration, facilitate replication, ensure transparency, and provide independent data analysis opportunities.

### Oversight and monitoring

#### Composition of the coordinating centre and trial steering committee {5d}

The trial will be overseen by a dedicated group of research coordinators and the Trial Steering Committee (TSC) to ensure its quality, integrity, and adherence to regulations. The research coordinators are responsible for day-to-day operations, including screening and consenting participants, managing data collection, and ensuring regulatory compliance. The TSC will provide oversight and will meet at least twice a year and more often if needed. This committee will comprise experienced trialists, a pediatric pharmacist, a health economist, a statistician, and the medical directors of the NICU and follow-up clinic.

#### Composition of the data monitoring committee, its role and reporting structure {21a}

The Data Monitoring Committee will comprise two independent members with expertise in neonatal research: Dr. Namasivayam Ambalavanan, MD, and Dr. Michael O’Shea, MD. Additionally, a qualified statistician, Claudia Pedroza, PhD, will be included to provide statistical expertise. They will review the interim analysis results when half the patients have reached 36 weeks’ PMA**Adverse event reporting and harms {22}**

The research team perform daily monitor for any adverse event related to excessive vitamin D intake. If hypercalcemia (serum Calcium >10.5 mg/dL or ionized Ca >1.55 mmol/L) is present, other laboratory parameters, such as phosphorus or magnesium, will be considered. It is common in this population to have high calcium levels with low phosphorus while in parenteral nutrition due to unadjusted calcium/phosphorus ratios. If that is the case, we will recommend modifications to the clinical team and monitor closely. If hypercalcemia occurs and other causes (e.g. low phosphorus) cannot be excluded, the NICU medical director will be contacted to determine if signs of vitamin D toxicity are found (nephrocalcinosis, trismus, feeding intolerance), stop the study drug, and notify the parents and IRB.

Due to the high mortality in the more immature infants in the study (22–25 weeks), only if unexpected or inexplicable death happens will it be communicated immediately to the IRB. If not, twice a year, a report of severe adverse events (deaths) will be shared with the IRB as part of the follow-up evaluation to allow the study to continue.

#### Frequency and plans for auditing trial conduct {23}

The study team will audit the data regularly. The IRB, the Food and Drug Administration, and the Office of Human Research Protections will have access to the study records per request.

#### Plans for communicating important protocol amendments to relevant parties (e.g. trial participants, ethical committees) {25}

The IRB will receive any protocol modifications, which will not occur until approval. The consent form will include the changes, and only the patients enrolled after the changes take place will be affected. ClinicalTrials.gov and sponsors will be notified of the changes.

#### Dissemination plans {31a}

Our parent advisory board will collaborate on a clear summary for study participants, sharing personalized letters via mail and a dedicated website. Healthcare professionals will access results on clinicaltrials.gov and the publication of the results in a reputable journal, through webinars and conferences. Public dissemination will be facilitated through social media and our study website, ensuring accessibility, and understanding for a lay audience.

## Discussion

While supplementation with up to 1000 IU/day may be used in many places, we chose 800 IU/day as the intervention arm because it is unlikely to cause toxicity even in EP infants [15]. A fixed rather than a weight-based dose is used because the smaller and more preterm the infant, the higher the risk for vitamin D deficiency, the longer it takes to reach full feedings [14, 36], and the greater the need for growth. Moreover, EP infants may also need a rapid increase in vitamin D levels after birth to optimize intracellular transport[10].

The needs for vitamin D and the metabolism in EP infants may differ from other populations [37, 38]. We will correlate the supplementation regimen, vitamin D metabolite levels analyzed by state-of-art, and clinical outcomes in EP infants. Our findings will help identify levels associated with the best clinical outcomes and avoid assuming that values considered normal in larger and older infants should be the goal values for EP infants [39, 40].

Our measure of respiratory support is ordinal. This avoids using a primary composite outcome (e.g., death or BPD), which gives equal statistical weight to outcomes of differing importance. Moreover, the ordinal outcome allows greater statistical power and a more meaningful assessment[41, 42]. Limitations of traditional frequentist analyses are increasingly recognized, and dichotomous conclusions based on arbitrary p-value thresholds are increasingly criticized [19, 20]. Bayesian analyses avoid these problems and allow direct assessment of the probability of treatment benefit – what clinicians, patients, and family members want to know – which is not obtainable with standard frequentist analyses [21, 22]. These advantages are particularly important in studies with limited sample size[23]. The posterior probability of benefit will be assessed using conservative neutral prior estimates with a 95% CrI within the range for clinical outcomes almost always found in major randomized trials (RR = 1.0; 95% CrI = 0.33–3.0]).

During our trial, we will survey different stakeholders (neonatologists, a random sample of neonatal bedside nurses, and the medical and nursing directors of our NICU) about potential barriers to adopting and implementing higher-dose vitamin D supplementation if our hypotheses are confirmed. This information will position our investigator team to have the largest and quickest impact on patient care after trial completion [43].

If our hypotheses are correct, the results may change clinical practice in conjunction with the supportive findings of prior or future trials. Even if a benefit is not shown, the study will advance the understanding of vitamin D metabolism in EP infants and help determine target 25-OH-D_3_ levels associated with the best clinical outcomes.

### Trial status

The protocol version number NCT05459298 is on ClinicalTrials.gov. The registration date was on July 14, 2022. Enrollment started on September 6, 2022 and enrollment to date is on schedule. The approximate date when recruitment will be completed on July 1, 2025.

## Figures and Tables

**Table 1:** Total vitamin D intake from all sources at various feeding intakes and times

Vitamin D intake	60 ml/kg/day of UF ≤28 days after birth	120 ml/kg/day of FF ≤28 days after birth	160 ml/kg/day FF ≤28 days after birth	160 ml/kg/day FF >28 days after birth
**Routine care**	~160 IU/kg/day from PN + <3 IU/day from UF* + Placebo	~140 IU/kg/day from FF +400IU/day** + Placebo	~190 IU/kg/day from FF+400IU/day + Placebo	~190 IU/kg/day from FF +400 IU/day
**Intervention**	160 IU/kg/day fromPN + <3 IU/day from UF* + 800 IU/d (study drug)	~140IU/kg/day from FF + 800 IU/d (study drug)	~190 IU/kg/day from FF + 800 IU/d (study drug)	~190 IU/kg/dayfrom FF + 400 IU/day

Parenteral nutrition [PN], unfortified feedings [UF], fortified feedings [FF]

* Breast milk vitamin D content is minimal (~3 IU/ 100 ml). Fortification starts when the infant tolerates feeds of at least 80 ml/kg/day

** 400 IU/day will not be started until the infant reaches 120ml/kg/day of breast milk with human milk fortifier up to 24 kcal/oz (~140 IU/kg/day)

**Table 2:** Ordinal respiratory outcomes

No BPD	Grade 1 BPD	Grade 2 BPD	Grade 3 BPD	Death
No respiratory support	NC≤2L/min	NC > 2 L/min, non-invasive ventilation	Intubated mechanical ventilation	Death before 36 weeks PMA

## Data Availability

The dataset to be collected and analyzed for this study will be available by contacting the corresponding author upon reasonable request.

## Abbreviations

25 OH-D_3_: 25-hydroxy vitamin D3
BSID IV: Bayley Scales of Infant and Toddler Development, fourth edition
BPD: Bronchopulmonary dysplasia
BW: Birth weight
CA: Corrected age
CrI: Credible Interval
EP: Extremely preterm
GA: Gestational age
IDP: Investigational drug pharmacy
IRB: Institutional Board Review
MCMC: Markov chain Monte Carlo
NDI: Neurodevelopmental impairment
NICU: Neonatal Intensive Care Unit
PMA: Postmenstrual age
REDCap: Research Electronic Data Capture
RR: Relative risk
SD: Standard deviation
ViDES: Vitamin D Extra Supplementation Trial
